# Assessment of methylene blue and NAD on LPS-induced acute brain injury: effects on neuroinflammation, oxidative stress, and blood–brain barrier integrity in a rat endotoxemia model

**DOI:** 10.1007/s00210-026-05451-1

**Published:** 2026-05-21

**Authors:** Songul Doganay, Hilal Üstündağ, Elif Erbaş, Sevinc Yanar, Serhat Hayme, Nezahat Kurt

**Affiliations:** 1 Department of Physiology, Faculty of Medicine, Sakarya University, Sakarya, Türkiye; 2 Department of Physiology, Faculty of Medicine, Erzincan Binali Yıldırım University, Erzincan, Türkiye; 3 Department of Histology and Embryology, Faculty of Veterinary Medicine, Atatürk University, Erzurum, Türkiye; 4 Department of Histology and Embryology, Faculty of Medicine, Sakarya University, Sakarya, Türkiye; 5 Department of Medical Biology, Faculty of Medicine, Ankara Yıldırım Beyazıt University, Ankara, Türkiye; 6 Department of Biostatistics and Health Informatics, Faculty of Medicine, Erzincan Binali Yıldırım University, Erzincan, Türkiye; 7 Department of Biochemistry, Faculty of Medicine, Erzincan Binali Yıldırım University, Erzincan, Türkiye

**Keywords:** Sepsis, Neuroinflammation, Methylene blue, Nicotinamide adenine dinucleotide (NAD), Blood–brain barrier, Oxidative stress, Combination therapy

## Abstract

Sepsis-induced brain dysfunction remains a significant challenge with limited therapeutic options, warranting exploration of multi-target approaches. Methylene blue (MET) is a phenothiazine derivative with mitochondrial-enhancing and electron transport chain regulatory properties, while nicotinamide adenine dinucleotide (NAD) serves as a crucial cofactor in cellular energy metabolism and redox reactions. This study investigated the therapeutic effects of MET and NAD, individually and in combination, in lipopolysaccharide (LPS)-induced sepsis model. Male Sprague–Dawley rats (*n* = 35) were randomized into five groups: control, LPS (8 mg/kg), LPS + NAD (250 mg/kg), LPS + MET (15 mg/kg), and LPS + NAD + MET. Brain tissues were analyzed for oxidative stress, inflammation, and blood–brain barrier integrity. The combination therapy demonstrated the most pronounced improvements across clinical parameters, antioxidant capacity evidenced by significantly elevated superoxide dismutase levels (*p* < 0.001) and cerebral histopathological outcomes. It also significantly reduced myeloperoxidase activity (*p* < 0.001) compared to the LPS group. Pro-inflammatory cytokines IL-6 and IL-8 were significantly decreased in both MET and NAD monotherapy groups (*p* < 0.05), while TNF-α showed a decreasing trend without statistical significance. Brain-derived neurotrophic factor levels were maintained in the combination group, comparable to controls, while TGF-β showed differential modulation across treatment groups. Immunohistochemical analysis revealed LPS-induced blood–brain barrier disruption, evidenced by reduced occludin expression (*p* < 0.01), increased aquaporin-4 expression in perivascular astrocytic end-feet (*p* < 0.05), and elevated glial fibrillary acidic protein reactivity in protoplasmic astrocytes (*p* < 0.01). In conclusion, MET and NAD individually provided significant protection against LPS-induced brain injury by reducing neuroinflammation, oxidative stress, and neuronal damage. The combined treatment offered incremental benefits in certain outcomes. These findings suggest that both agents may have therapeutic potential in SAE.

## Introduction

Despite advancements in critical care medicine, sepsis remains a potentially fatal condition characterized by an abnormal host response to infection that can lead to multiple organ failure and high mortality (Hotchkiss et al. 2016; Ustundag 2026; Borges and Bento 2024). Among the organs affected, the brain is particularly vulnerable to sepsis-induced dysfunction, with up to 70% of septic patients developing neurological complications ranging from delirium to long-term cognitive impairment (Sekino et al. 2022). The economic and social burden of sepsis-associated brain dysfunction is substantial, with survivors often experiencing persistent cognitive deficits that reduce quality of life and increase healthcare costs (Iwashyna et al. 2010).

The pathophysiology of sepsis-induced brain dysfunction involves complex interactions between systemic inflammation, oxidative stress, mitochondrial dysfunction, and blood–brain barrier disruption (Liu et al. 2025). In sepsis, bacterial components like lipopolysaccharide from gram-negative bacteria interact with host pattern recognition receptors, initiating inflammatory cascades that result in the production of cytokines such as TNF-α, IL-1β, and IL-6 (Rajaee et al. 2018). These endotoxins trigger widespread immune activation, leading to dysregulated cytokine release and endothelial dysfunction that compromises the blood–brain barrier (Peng et al. 2021). Cytokines reach the brain through areas with reduced blood–brain barrier integrity and via vagus nerve activation, leading to neuroinflammation (Sun et al. 2022). This neuroinflammatory response involves microglial activation, astrogliosis, and reactive oxygen species (ROS) production, which collectively contribute to neuronal damage and synaptic dysfunction (Moraes et al. 2021; Xin et al. 2023). Additionally, sepsis induces mitochondrial dysfunction in brain tissue, leading to energy depletion, increased ROS production, and activation of apoptotic pathways (Liu et al. 2024).

Blood–brain barrier disruption has emerged as a central mechanism in sepsis-associated encephalopathy. During sepsis, pro-inflammatory mediators and oxidative stress compromise blood–brain barrier integrity by disrupting tight junction proteins (Barichello et al. 2021). Recent investigations demonstrated real-time alterations in blood–brain barrier permeability in experimental sepsis models, correlating with neurological dysfunction severity (Kuperberg and Wadgaonkar 2017). Specific tight junction proteins, including occludin and zonula occludens-1, are particularly vulnerable to sepsis-induced dysregulation, providing potential therapeutic targets (Ni et al. 2019).

Current treatment approaches for sepsis-associated brain dysfunction remain largely supportive, focusing on controlling underlying infection, maintaining cerebral perfusion, and avoiding neurotoxic medications (Pan et al. 2022). Available therapeutic interventions include dexmedetomidine, which provides neuroprotection through α2-adrenergic receptor activation but carries risks of bradycardia and prolonged sedation (Mei et al. 2021). Emerging therapies such as melatonin have demonstrated neuroprotective effects by restoring hippocampal BDNF expression and controlling oxidative stress (Ji et al. 2018). However, these therapies predominantly target single pathophysiological mechanisms. Clinical trials evaluating anti-inflammatory agents, antioxidants, and other neuroprotective strategies have yielded inconsistent results, highlighting the need for multi-target approaches (Mallah et al. 2020).

Methylene blue (MET), a phenothiazine derivative used clinically for methemoglobinemia, has demonstrated neuroprotective effects through its ability to improve mitochondrial function, reduce oxidative stress, and inhibit nitric oxide synthase (Gureev et al. 2022). In experimental stroke, MET reduced infarct volume by preserving mitochondrial function. Studies in traumatic brain injury showed MET decreased neuroinflammation and improved cognitive recovery through electron transport chain modulation (Fenn et al. 2015). Tucker et al. reported that MET administration in sepsis models preserved mitochondrial membrane potential and reduced neuronal apoptosis (Tucker et al. 2018). Similarly, nicotinamide adenine dinucleotide (NAD), a critical coenzyme in cellular metabolism, has gained attention for its role in regulating oxidative stress, inflammation, and cellular bioenergetics (Xie et al. 2020). Hong et al. demonstrated that NAD supplementation enhanced cellular resilience to stress through activation of sirtuin-dependent pathways (Hong et al. 2018), while She et al. found that nicotinamide riboside exerted neuroprotective effects against intracerebral hemorrhage by inhibiting neuroinflammation and oxidative stress (She et al. 2025).

Despite extensive evidence supporting individual neuroprotective effects of MET and NAD, their combined potential in sepsis-induced brain dysfunction remains unexplored. The present study investigated the neuroprotective effects of MET and NAD, individually and in combination, in an LPS-induced rat sepsis model. We hypothesized their combination would provide superior protection against neuroinflammation, oxidative stress, and blood–brain barrier disruption. Using clinical scoring, biochemical analyses, and immunohistochemical evaluations, we assessed their effects on sepsis-induced brain injury to develop potential multi-target neuroprotective strategies.

## Materials and methods

### Reagents

Lipopolysaccharide (LPS) from *Escherichia coli* O111 and NAD (Item no; N0636) were purchased from Sigma-Aldrich (St. Louis, MO, USA), while methylene blue (MET) was obtained from Vem Pharmaceutical Industry (Sariyer, İstanbul, Turkey). Ketamine hydrochloride (Ketalar®) was obtained from Pfizer (New York, NY, USA) and xylazine (Rompun®) from Bayer (Leverkusen, Germany). For biochemical analyses, commercial ELISA kits for MPO (Cat No. E574Ra, BT LAB), SOD (Cat No. E0168Ra, BT LAB), IL-6 (Cat No. ELK1158, ELK Biotechnology), TNF-α (Cat No. ELK1396, ELK Biotechnology), IL-8 (Cat No. E1167Ra, BT LAB), BDNF (Cat No. E0476Ra, BT LAB), TGF-β (Cat No. ELK2311, ELK Biotechnology), and NF-κB (Cat No. 201–11–5141, SunRed) were used. For immunohistochemical analyses, primary antibodies against AQP-4 (BT LAB, BT-AP00558), GFAP (Bioss, bs-0199R), and Occludin (Affinity, catalog no: Ab-BF8330) were used. Secondary antibody kit (Large Volume Detection System: anti-Polyvalent, catalog no: TP-125-HL) and DAB (3,3′-Diaminobenzidine) were obtained from Thermo Fisher Scientific (Waltham, MA, USA).

### Animals and experimental design

The research protocol received approval from Sakarya University's Animal Ethics Committee and adhered to the National Institutes of Health guidelines for laboratory animal care. The study utilized 35 male Sprague–Dawley rats (aged 10–12 weeks, weight range 250–280 g) sourced from Sakarya University Experimental Animals Research Center. The study strictly adhered to the ARRIVE guidelines and was conducted following the national regulations for the ethical use and care of laboratory animals. The animals were maintained in polycarbonate enclosures (4–5 rats per cage) under regulated environmental parameters (22 ± 2 °C, 50–60% humidity, alternating 12-h light/dark periods) with unrestricted access to standard feed and water. A one-week acclimatization period preceded experimental procedures. Sample size was determined using G*Power software (version 3.1.9.7, Heinrich-Heine-Universität Düsseldorf, Germany) with *α* = 0.05, power (1-*β*) = 0.8, and effect size *f*= 0.65 based on previous similar studies examining oxidative stress parameters in sepsis models. The calculation indicated that a minimum of 6 animals per group was required (Kara and Ozkanlar 2023). We assigned 7 animals per group to account for potential losses.

### Experimental groups

After acclimatization, rats were randomly assigned to five experimental groups (*n* = 7 each):

- Group 1 (Control): Rats received a single intraperitoneal (i.p.) injection of sterile saline (0.9% NaCl, equal volume to LPS injection).
- Group 2 (LPS): Rats received a single i.p. injection of LPS (8 mg/kg) (Yakut et al. 2024).
- Group 3 (LPS + NAD): Rats received i.p. LPS (8 mg/kg) followed by i.p. NAD (250 mg/kg) (Guo et al. 2021) 1 h later. NAD was dissolved in sterile saline to obtain a concentration of 100 mg/ml.
- Group 4 (LPS + MET): Rats received i.p. LPS (8 mg/kg) followed by i.p. MET (15 mg/kg, 1% solution) (Collange et al. 2013) 1 h later. Methylene blue was prepared as a 1% solution (10 mg/ml) in sterile saline.
- Group 5 (LPS + NADMET): Rats received i.p. LPS (8 mg/kg) followed by both i.p. NAD (250 mg/kg) and i.p. MET (15 mg/kg, 1% solution) 1 h later sequentially with a 5-min interval between injections.

The dosage of LPS, NAD^+^, and MET was determined according to previous experiments.

### Murine Sepsis Score (MSS) assessment

The severity of sepsis and treatment efficacy were evaluated using a modified MSS as described by Shrum et al. (Shrum et al. 2014). Animals were assessed at baseline and at 2, 4, and 6 h post-LPS injection by two blinded observers who scored seven clinical parameters: appearance, level of consciousness, activity, response to stimulus, eye condition, respiration rate, and respiration quality. Each parameter was scored from 0 (normal) to 4 (severe), with excellent inter-observer reliability (*κ* = 0.87). The total MSS score (0–28) was calculated as the sum of all parameters, with higher scores indicating more severe sepsis. This standardized scoring system enabled objective assessment of disease progression and treatment response throughout the experimental period, with no animals reaching the humane endpoint criteria (score ≥ 24 or unresponsiveness) during the study.

### Sample collection

Following LPSadministration by 6 h (Yakut et al. 2024), the rats underwent anesthesia using a ketamine/xylazine combination (80/5 mg/kg, administered intraperitoneally). Cardiac puncture facilitated blood collection for serum preparation. Under deep anesthesia, the animals were euthanized by exsanguination. The extracted brain tissue was cleansed with cold phosphate-buffered saline (pH 7.4) before sagittal division. The right hemispheric portion was aliquoted into small volumes, rapidly frozen in liquid nitrogen, and then stored at − 80 °C until biochemical analysis which was conducted using ice-cold buffers and performed on ice, to prevent any thaw-related physiological changes during processing. The left hemisphere was preserved in neutral buffered formalin (10%) for 72 h to facilitate histological and immunohistochemical analysis.

### Biochemical analyses

#### Tissue homogenization

Frozen whole brain tissue samples were thawed and homogenized in ice-cold PBS (1:10 w/v) using a tissue homogenizer at 5000 rpm for 2 min. The homogenates were centrifuged at 10,000 × g for 15 min at 4 °C, and the supernatants were collected and stored at − 80 °C until analysis. All samples were coded prior to analysis, and biochemical measurements were performed by personnel blinded to group assignments.

#### Oxidative stress and antioxidant parameters

Brain tissue samples were homogenized and protein content was determined using the Bradford technique (Kruger 2002) with bovine serum albumin as reference. SOD activity was measured using the Rat Super Oxidase Dismutase ELISA Kit (Cat No. E0168Ra, BT LAB) and MPO activity was determined using the Rat Myeloperoxidase ELISA Kit (Cat No. E574Ra, BT LAB). Measurements were performed according to the manufacturer’s instructions, with tissue samples, standard samples, and streptavidin-HRP added to microplate wells coated with rat MPO and SOD-specific monoclonal antibodies, followed by incubation at 37 °C for 60 min. After washing, chromogenic reagent was added and plates were incubated at 37 °C for 10 min. Stop solution was then added and absorbance was measured at 450 nm. MPO and SOD standard curves were used for calculating absorbance values.

#### Inflammatory and neuroprotective markers

All inflammatory markers (TNF-α, IL-6, IL-8, and NF-κB) and neuroprotective markers (BDNF) were measured in brain tissue homogenates using the same tissue preparation protocol described for oxidative stress parameters. Brain tissue TNF-α, IL-6, IL-8, NF-κB, and BDNF levels were measured using the rat ELISA kits. All measurements were performed according to the manufacturer’s instructions following the same microplate protocol described for oxidative stress parameters, with tissue homogenates, standard samples, and streptavidin-HRP added to antibody-coated wells, incubated at 37 °C for 60 min, followed by chromogenic reagent addition and absorbance measurement at 450 nm.

### Histopathological and immunohistochemical analyses

#### Histopathological examination

During systemic necropsy, brain tissues from all experimental groups were extracted and immediately fixed in 10% neutral buffered formalin for a 72-h period. The fixed specimens underwent progressive dehydration through an ethanol gradient (50% through 100%), xylene clearing, and paraffin embedding. A rotary microtome was employed to create 5-μm sections that were subsequently mounted on glass slides. Serial sections were obtained from each brain tissue sample with three replicates per animal. The prepared sections received Mallory’s trichrome stain with Crossman’s modification before examination and photographic documentation under a modified light microscope imaging system at 200 × magnification. Twenty different microscopic fields were photographed from each section to ensure representative sampling across cortical regions.

### Neuronal damage scoring

Neuronal damage in the brain tissue was assessed by evaluating five randomly selected areas of the left cerebral cortex from each animal. Neuronal injury was identified by the presence of hypereosinophilia in cell morphology, cytoplasmic shrinkage, and nuclear pyknosis. All histopathological assessments were performed by a blinded observer to eliminate bias. Two independent time-point evaluations were conducted for each experimental group to ensure reproducibility, and the consistency of results was confirmed. The extent of neuronal injury was quantified using a semiquantitative scale based on the proportion of damaged neurons relative to the total cell count: 0 = 0%, 1 = 1–20%, 2 = 20–40%, 3 = 40–60%, 4 = 60–80%, and 5 = 80–100% (Veldeman et al. 2017). Final scores were calculated as the mean of all evaluated fields.

### Immunohistochemical analysis

For immunohistochemical staining, tissue sections were mounted on positively charged slides and incubated at 60 °C in a drying oven for 1 h. Subsequently, sections were deparaffinized in two separate xylene baths for 15 min each. Following deparaffinization, the sections were rehydrated through a descending ethanol series (100%, 96%, 80%, and 70%), spending 5 min in each solution, then rinsed with distilled water to proceed to immunohistochemical staining.

The antigen retrieval process involved heating the slides in citrate buffer solution (pH 6.0) in a 600-W microwave oven through four 5-min cycles. After allowing the slides to reach ambient temperature, phosphate-buffered saline rinsing was performed before a 10-min dark incubation in 3% hydrogen peroxide solution to neutralize endogenous peroxidase activity. Following another phosphate-buffered saline wash, protein blocking agent was applied for 5 min.

The sections were incubated overnight at 4 °C with primary antibodies: anti-AQP-4 (BT LAB, BT-AP00558, 1:100 dilution), anti-GFAP (Bioss, bs-0199R, 1:100 dilution), anti-Caspase-3 (Cell Signaling Technology, CST-9664, 1:200 dilution), and anti-Occludin (Affinity, Ab-BF8330, 1:100 dilution). After washing in PBS, secondary antibody and HRP conjugate (Thermo Fisher, TP-125-HL) were applied sequentially for 30 min each, with PBS rinsing between steps. Reagents were applied according to the manufacturers’ protocols.

The slides were developed using DAB (3,3′-diaminobenzidine; Thermo Fisher) as the chromogen, counterstained with Mayer’s hematoxylin, and coverslipped with Entellan. The immunohistochemically stained sections were examined and photographed under a light microscope (Nikon Eclipse E600) at 200 × magnification.

Staining intensity was evaluated in five microscopic fields and scored by a histopathologist according to the following criteria: 0 = 0%, 1 = 1–33%, 2 = 34–66%, and 3 ≥ 66% positive staining (Soliman et al. 2013).

### Statistical analysis

Data were analyzed using IBM SPSS 25.0 (SPSS Inc., Chicago, IL, USA). Normality was assessed by the Shapiro–Wilk test on 1000 bootstrap samples, and homogeneity of variances by Levene’s test. Biochemical parameters were compared using one-way ANOVA (with LSD post-hoc test) or Welch ANOVA (with Games-Howell post-hoc test) where appropriate; these data are presented as mean ± SEM. Histopathological and immunohistochemical scores were analyzed using one-way ANOVA followed by Tukey’s post-hoc test and are presented as mean ± SD. Sample size was *n* = 7 per group for all analyses. Statistical significance was set at *p* < 0.05 and indicated by asterisks (*p < 0.05, **p < 0.01, and ***p < 0.001).

## Results

### Overview of experimental animals and study completion

All 35 Sprague Dawley rats completed the experimental protocol with no dropouts or exclusions. Following LPS administration, animals in the LPS group exhibited typical sepsis-associated clinical signs including reduced activity, piloerection, lethargy, and diminished response to stimuli, while control animals maintained normal behavior and appearance. No unexpected adverse events or deviations from the planned methodology occurred during the study period. The severity of clinical manifestations varied significantly between treatment groups as detailed in Table 1.

**Table 1:** Comparison of Murine Sepsis Score (MSS) parameters among control and treatment groups

Variable	Score and description	Control (median [min–max])	LPS (median [min–max])	LPS + NAD (median [min–max])	LPS + MET (median [min–max])	LPS + NAD + MET (median [min–max])	*p*-value
Appearance	0: Smooth fur 1: Local piloerection 2: Widespread piloerection on back 3: “Puffed” appearance 4: Obvious weakness	0.00 [0–0]	3.00 [2–4]^a^	2.00 [1–3]^b^	2.00 [1–3]^b^	1.00 [0–2]^c^	< 0.05
Level of consciousness	0: Active 1: Avoids standing 2: Slow movement 3: Moves only when provoked 4: Immobile	0.00 [0–0]	4.00 [3, 4]^a^	2.00 [1–3]^b^	3.00 [2–4]^b^	1.00 [1, 2]^c^	< 0.05
Activity	0: Normal activity 1: Mildly suppressed 2: Occasional movement 3: Immobile 4: Tremorous immobility	0.00 [0–0]	4.00 [3, 4]^a^	2.00 [2]^b^	3.00 [3]^c^	1.00 [1, 2]^c^	< 0.05
Response to stimulus	0: Immediate response 1: Slow response 2: Partial response to touch 3: Minimal response 4: No response	0.00 [0–0]	4.00 [3, 4]^a^	2.00 [2]^b^	3.00 [2, 3]^b^	1.00 [1, 2]^c^	< 0.05
Eyes	0: Open 1: Partially closed 2: Half-closed 3: Swelling/secretions 4: Fully closed/cloudy	0.00 [0–1]	3.00 [3, 4]^a^	2.00 [2]^b^	2.00 [2]^b^	1.00 [1, 2]^c^	< 0.05
Respiration rate	0: Normal 1: Slightly reduced 2: Significantly reduced 3: Severely reduced 4: Very sparse	0.00 [0–0]	3.00 [3, 4]^a^	2.00 [1, 2]^b^	2.00 [2, 3]^b^	1.00 [1, 2]^c^	< 0.05
Respiration quality	0: Normal 1: Occasional straining 2: Continuous straining 3: Intermittent gasping 4: Continuous gasping	0.00 [0–1]	3.00 [3, 4]^a^	1.00 [1]^b^	2.00 [2]^b^,	1.00 [1, 2]^c^	< 0.05
Total MSS Score		0.00 [0–2]	24.00 [20–28]^a^	13.00 [10–15]^b^	17.00 [14–19]^b^	7.00 [5–10]^c^	< 0.05

The *p*-values in the table represent the results of pairwise comparisons between groups. A *p*-value < 0.05 indicates a statistically significant difference between the groups. The Mann–Whitney U test was used for pairwise comparisons, and the Bonferroni correction was applied. **Symbols (a, b, c):** These symbols indicate statistically significant differences between groups: **a;**significant difference compared to the Control group (*p* < 0.05); **b;**significant difference compared to the LPS group (*p* < 0.05); and **c;**significant difference compared to the LPS + NAD and LPS + MET groups (*p* < 0.05)

*Control* Control group, *LPS* LPS group, *LPS* + *NAD* LPS and NAD combination, *LPS* + *MET* LPS and MET combination, *LPS* + *NAD* + *MET* LPS, NAD, and MET combination

### Effects of treatments on clinical sepsis parameters

As shown in Table 1, LPS administration induced severe clinical manifestations of sepsis across all assessed parameters. Compared to the control group, LPS-treated animals showed significantly impaired appearance (*p* < 0.05), consciousness (*p* < 0.05), activity (*p* < 0.05), and response to stimuli (*p* < 0.05). Treatment with NAD, MET, or their combination significantly improved these clinical parameters compared to the LPS group. The combination therapy (LPS + MET + NAD) demonstrated significant improvement in clinical parameters, with lower MSS scores compared to individual treatments (LPS + NAD or LPS + MET) (p < 0.05).

### Effects on inflammatory cytokines and NF-κB activation

**Fig. 1:**
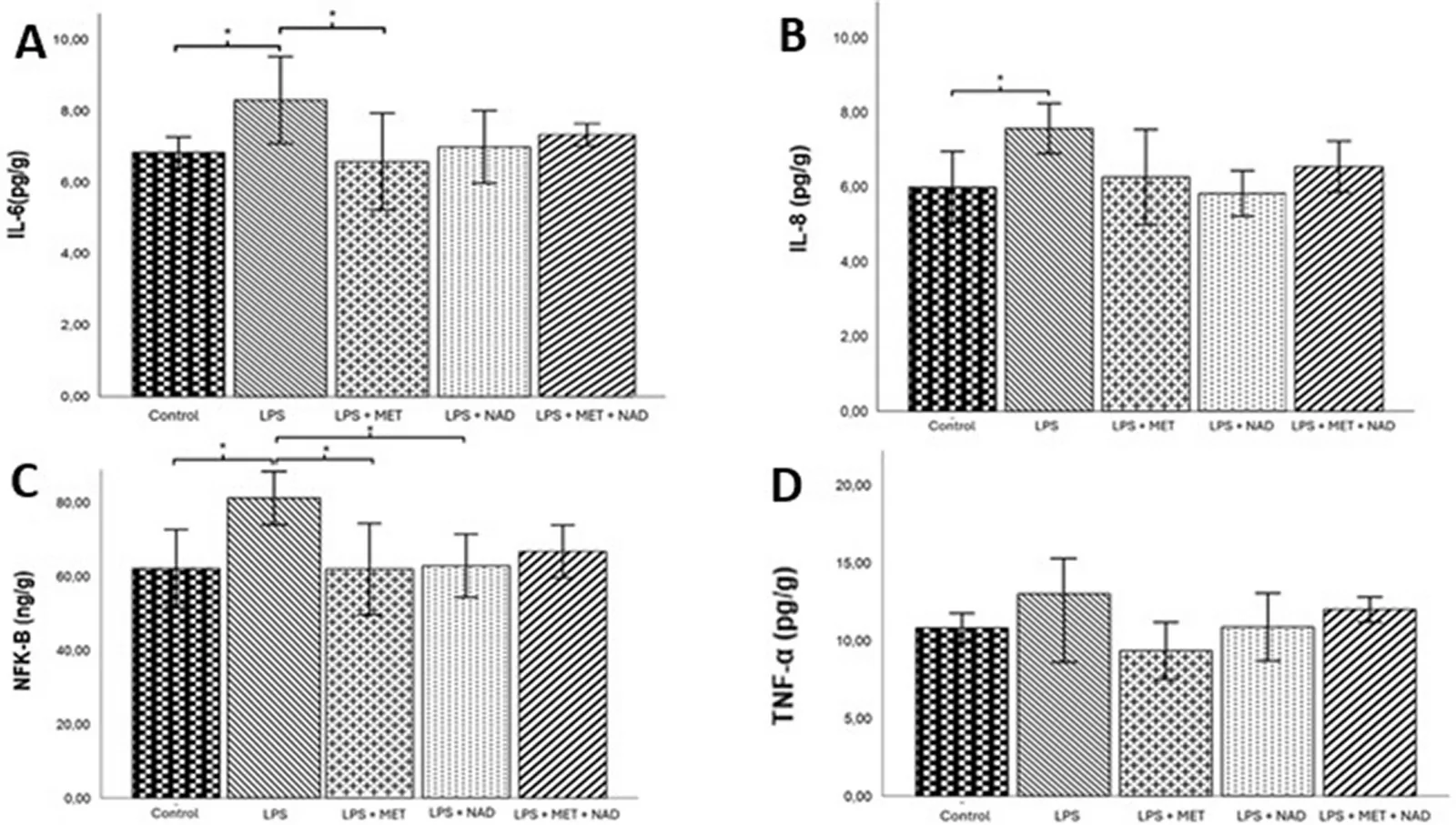
Effects of methylene blue (MET) and nicotinamide adenine dinucleotide (NAD) on inflammatory cytokines and NF-κB levels in LPS-induced sepsis model. Bar graphs showing the concentrations of **A:** IL-6, **B:** IL-8, **C:** NF-κB, and **D:** TNF-α in brain tissue across experimental groups. The data has been presented as mean ± SEM (*n* = 7 per group). Statistical significance is indicated by asterisks (*p < 0.05)

LPS administration significantly increased pro-inflammatory cytokine levels in brain tissue. IL-6 levels were significantly elevated in the LPS group compared to controls (*p* < 0.05) (Fig. 1). Individual treatments with either MET or NAD significantly reduced IL-6 levels (p < 0.05) compared to the LPS group. The combination therapy (LPS + MET + NAD) showed decreased IL-6 levels compared to the LPS group, although this reduction did not reach statistical significance. Similar patterns were observed for IL-8 levels (Fig. 1), with significant elevation in the LPS group compared to controls (*p* < 0.05). All treatments reduced IL-8 levels compared to the LPS group (*p* > 0.05). NF-κB, a key transcription factor mediating inflammatory responses, showed significant activation in the LPS group compared to controls (*p* < 0.05) (Fig. 1). Both individual treatments significantly reduced NF-κB levels (*p* < 0.05) to values comparable to controls. The combination therapy also reduced NF-κB activation compared to the LPS group, though not as prominently as the individual treatments. Regarding TNF-α, although a trend toward increased levels was observed in the LPS group compared to controls, this difference did not reach statistical significance (*p* > 0.05). All treatment groups showed TNF-α levels comparable to the control group.

### Effects on oxidative stress and antioxidant parameters

**Fig. 2:**
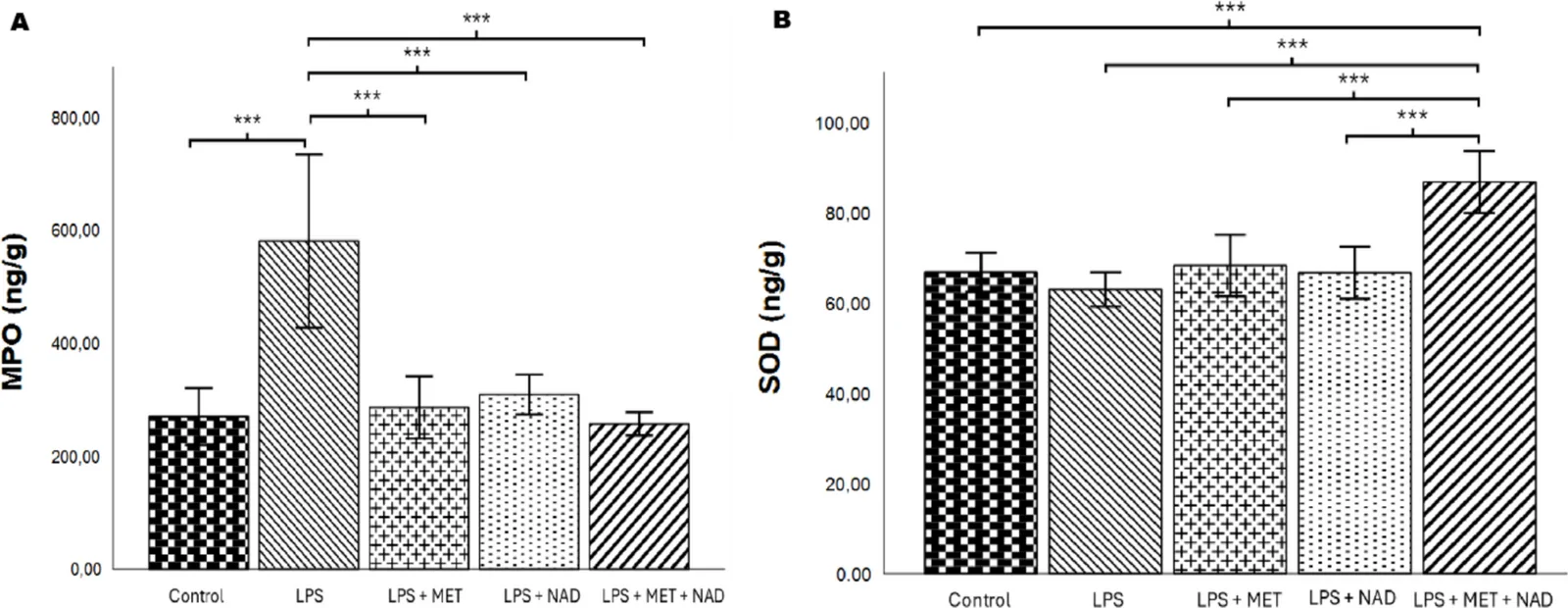
Comparison of MPO activity and SOD levels across experimental groups. **A:** MPO activity. **B:** SOD levels. The data has been presented as mean ± SEM (*n* = 7 per group). Statistical significance is indicated by asterisks (***p < 0.001)

LPS administration significantly increased MPO activity in brain tissue compared to the control group (*p* < 0.001), indicating enhanced neutrophil infiltration and oxidative stress (Fig. 2A). All treatment regimens significantly reduced MPO activity compared to the LPS group (*p* < 0.001), with the combination therapy (LPS + MET + NAD) showing the most reduction restoring levels comparable to the control group.

As shown in Fig. 2B, while LPS alone had minimal impact on SOD levels compared to controls, the combination therapy significantly elevated SOD activity (*p* < 0.001), demonstrating a superior antioxidant response compared to either NAD or MET alone. This enhanced antioxidant capacity with combination therapy represents a significant finding, suggesting an additive effect of NAD and MET in boosting endogenous antioxidant defenses during sepsis.

### Effects on neuroprotective factors

**Fig. 3:**
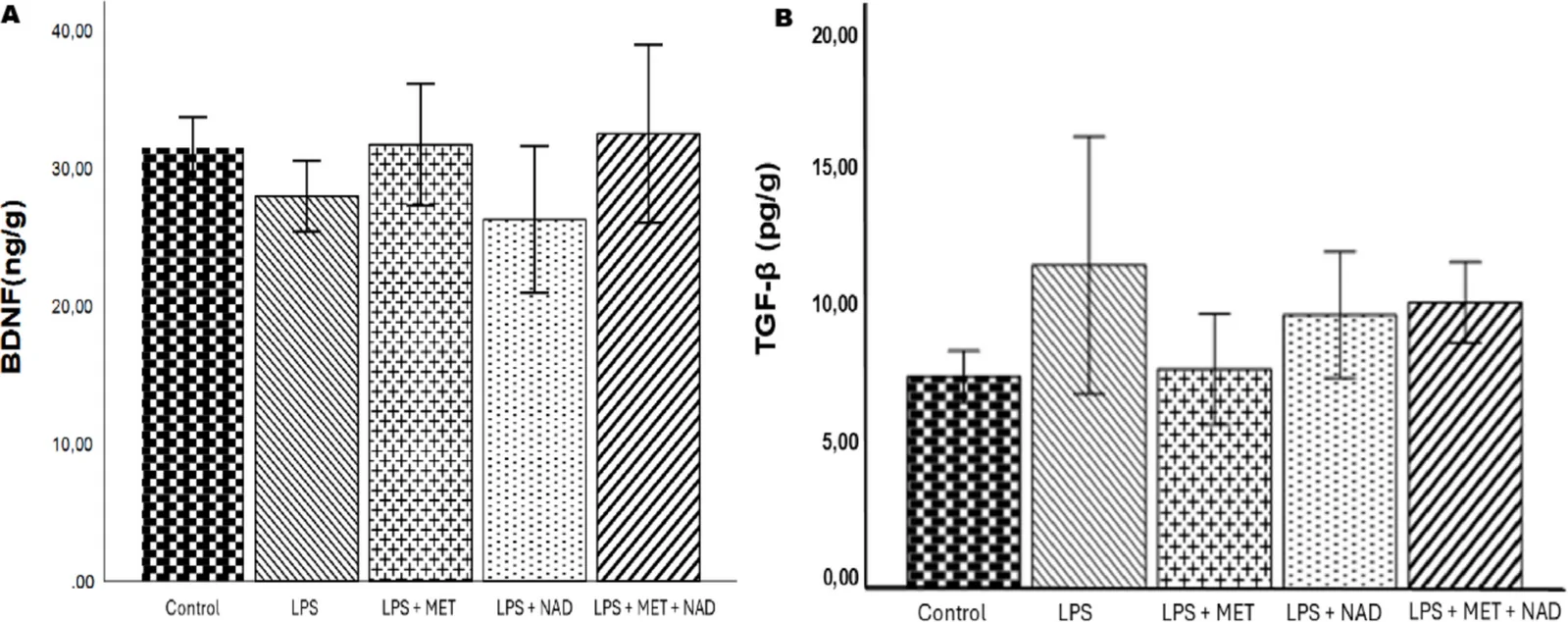
Effects of methylene blue and NAD on BDNF and TGF-β levels in brain tissue. **A:** BDNF levels. **B:** TGF-β levels. The data has been presented as mean ± SEM (*n* = 7 per group)

BDNF, an important neurotrophin promoting neuronal survival, showed a tendency to decrease in the LPS group compared to controls, although this difference was not statistically significant (*p* > 0.05) (Fig. 3A). Treatment with MET alone or the combination therapy maintained BDNF levels comparable to controls, suggesting a potential neuroprotective effect. NAD treatment alone did not significantly improve BDNF levels compared to the LPS group.

TGF-β levels (Fig. 3B) were increased in the LPS group compared to controls, although statistical significance was not reached (*p* > 0.05). The treatment groups showed intermediate values, suggesting a modulating effect on this anti-inflammatory cytokine.

### Histopathological findings

**Fig. 4:**
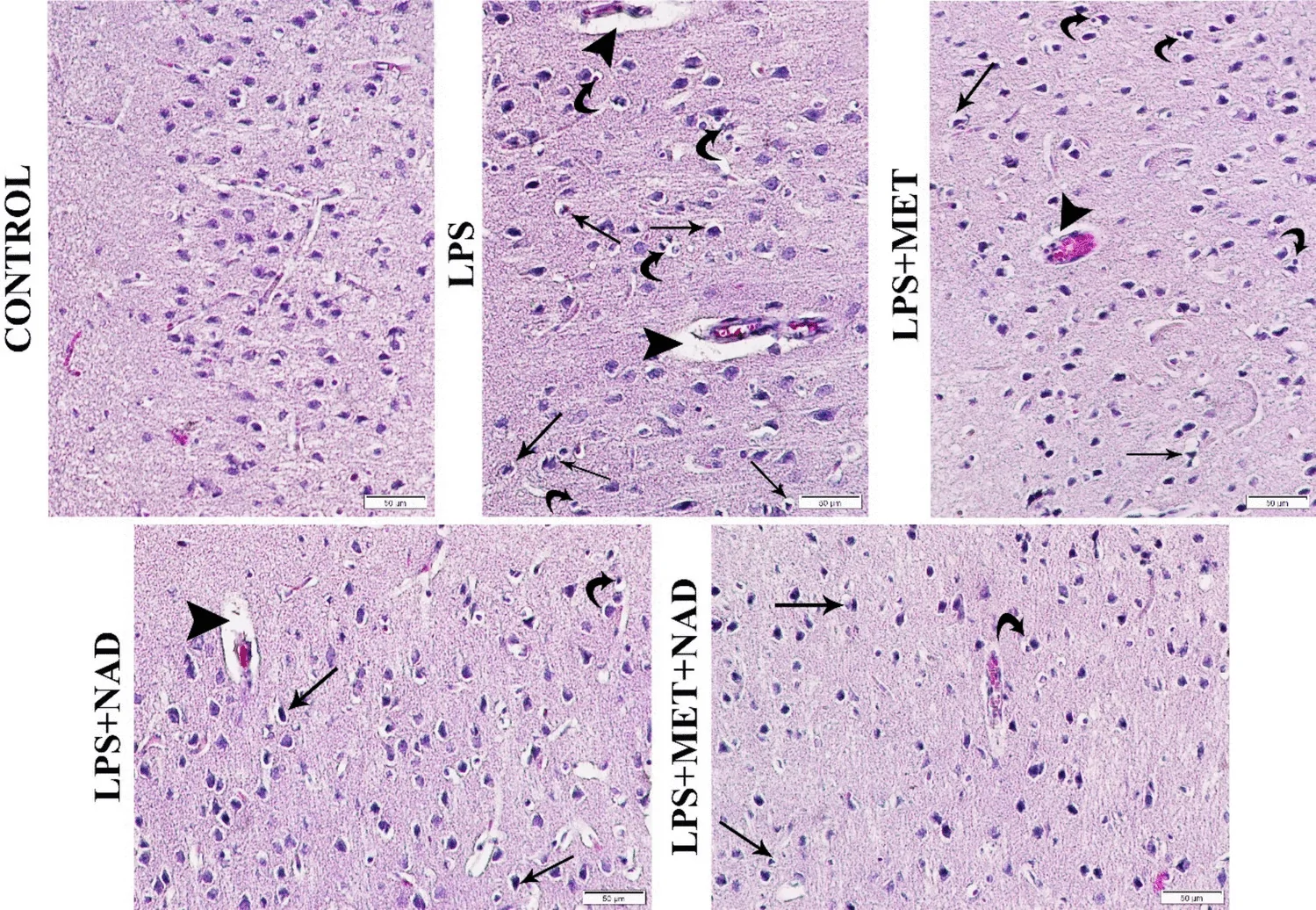
Photomicrographs of cerebral cortex histopathology in rat brain tissue (Mallory’s trichrome stain modified by Crossman, 200× magnification, scale bar 50 μm). Control group shows normal histology, while the LPS group exhibits marked neuronal damage characterized by perivascular dilation (arrowheads), degenerated neurons with pyknotic nuclei and perineuronal vacuolization (arrows), and microglial cell proliferation with satellitosis (curved arrow)

**Fig. 5:**
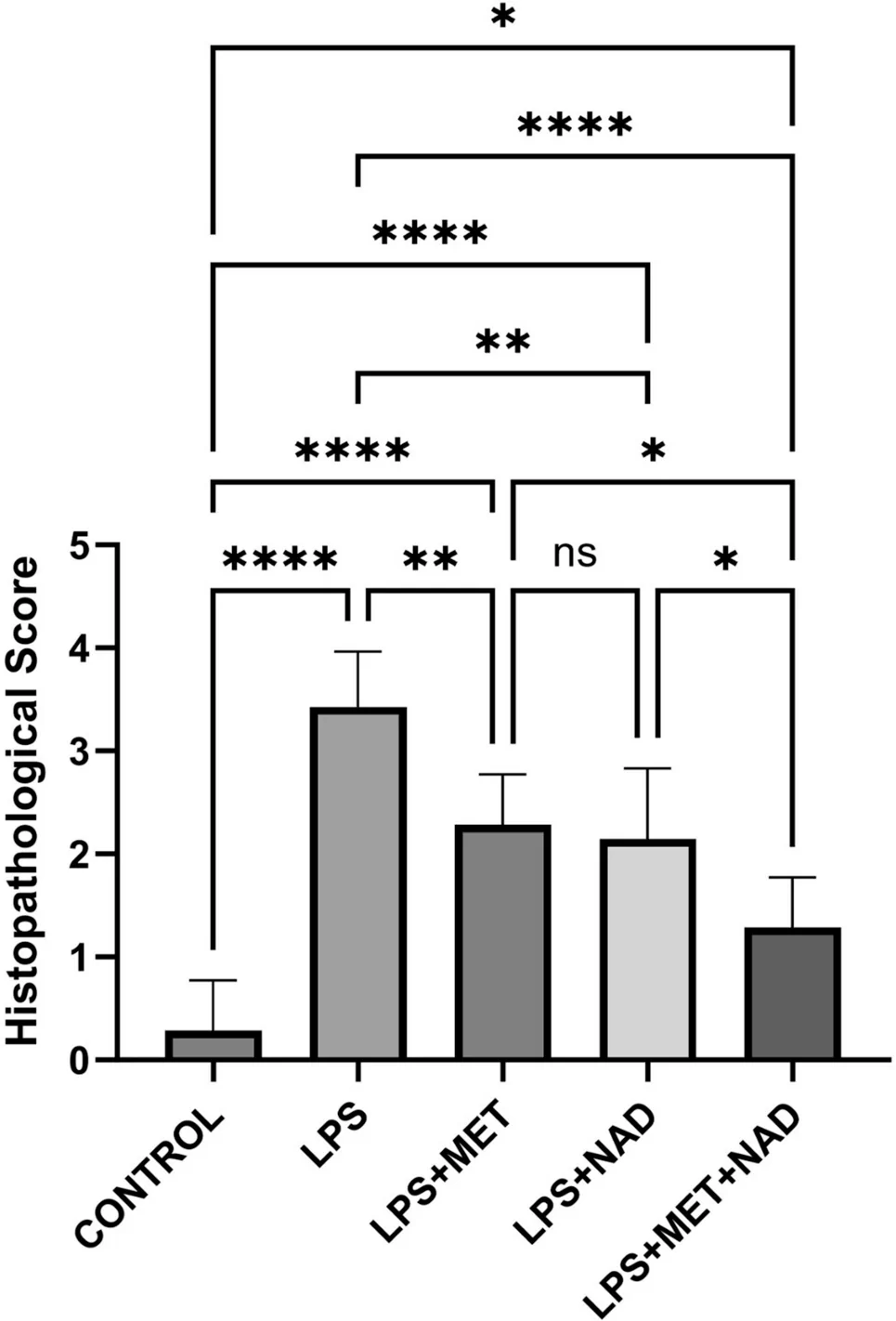
Histopathological scoring of brain tissue across all experimental groups. The graph shows quantitative assessment of neuronal damage based on semi-quantitative scoring criteria. Values are presented as mean ± SD (*n* = 7) and analyzed using one-way ANOVA followed by Tukey’s test. Asterisks indicate statistically significant differences between groups (*p = 0.01, **p = 0.001, and ****p < 0.0001)

Histopathological examination of brain tissue revealed significant differences between the experimental groups. The control group exhibited normal brain histology with no pathological findings. In contrast, the LPS group showed substantial neuronal damage in the cerebral cortex characterized by neuronal necrosis with nuclear pyknosis, perineuronal vacuolization, mild vascular congestion, neuronophagy, and satellitosis (proliferation of glial cells around damaged neuronal cell bodies). Both treatment groups (LPS + MET and LPS + NAD) showed markedly reduced histopathological lesions compared to the LPS group. The combination therapy group (LPS + MET + NAD) demonstrated notable improvement, with reduced histopathological alterations compared to monotherapy groups. These findings are illustrated in Fig. 4 and quantitatively represented in Fig. 5.

### Immunohistochemical analyses

**Fig. 6:**
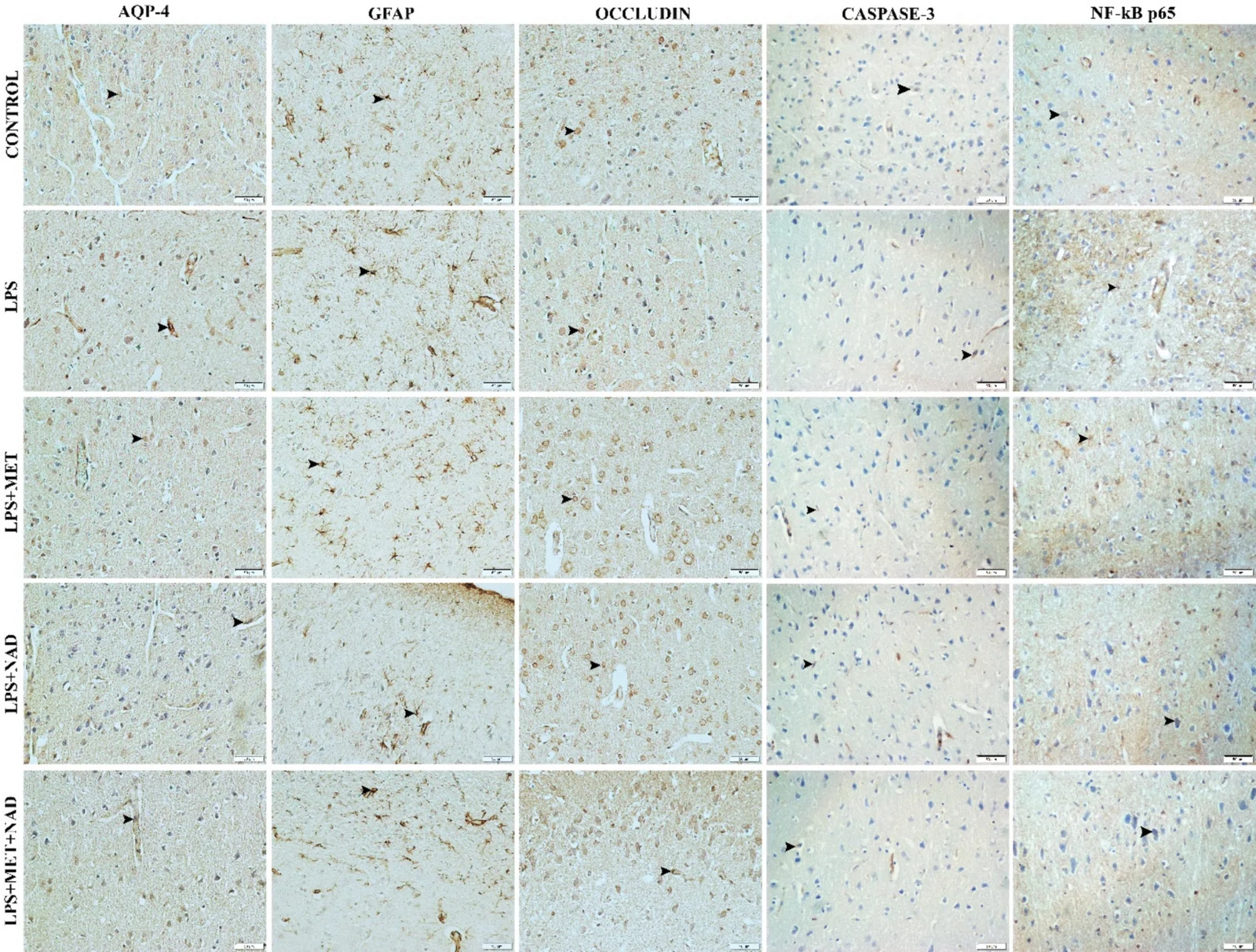
Immunohistochemical staining for AQP-4, GFAP, occludin, cleaved caspase-3, and NF-κB p65 in brain tissue across control, LPS, LPS + MET, LPS + NAD, and LPS + MET + NAD groups (Streptavidin–Biotin Peroxidase Method, 200 × magnification). Positively stained cells (arrowheads) are visualized by brown DAB chromogen precipitation. Note the increased AQP-4 and GFAP immunoreactivity in the LPS group compared to control, decreased occludin expression in the LPS group, and variable patterns of cleaved Caspase-3 and NF-κB p65 staining across treatment groups

**Fig. 7:**
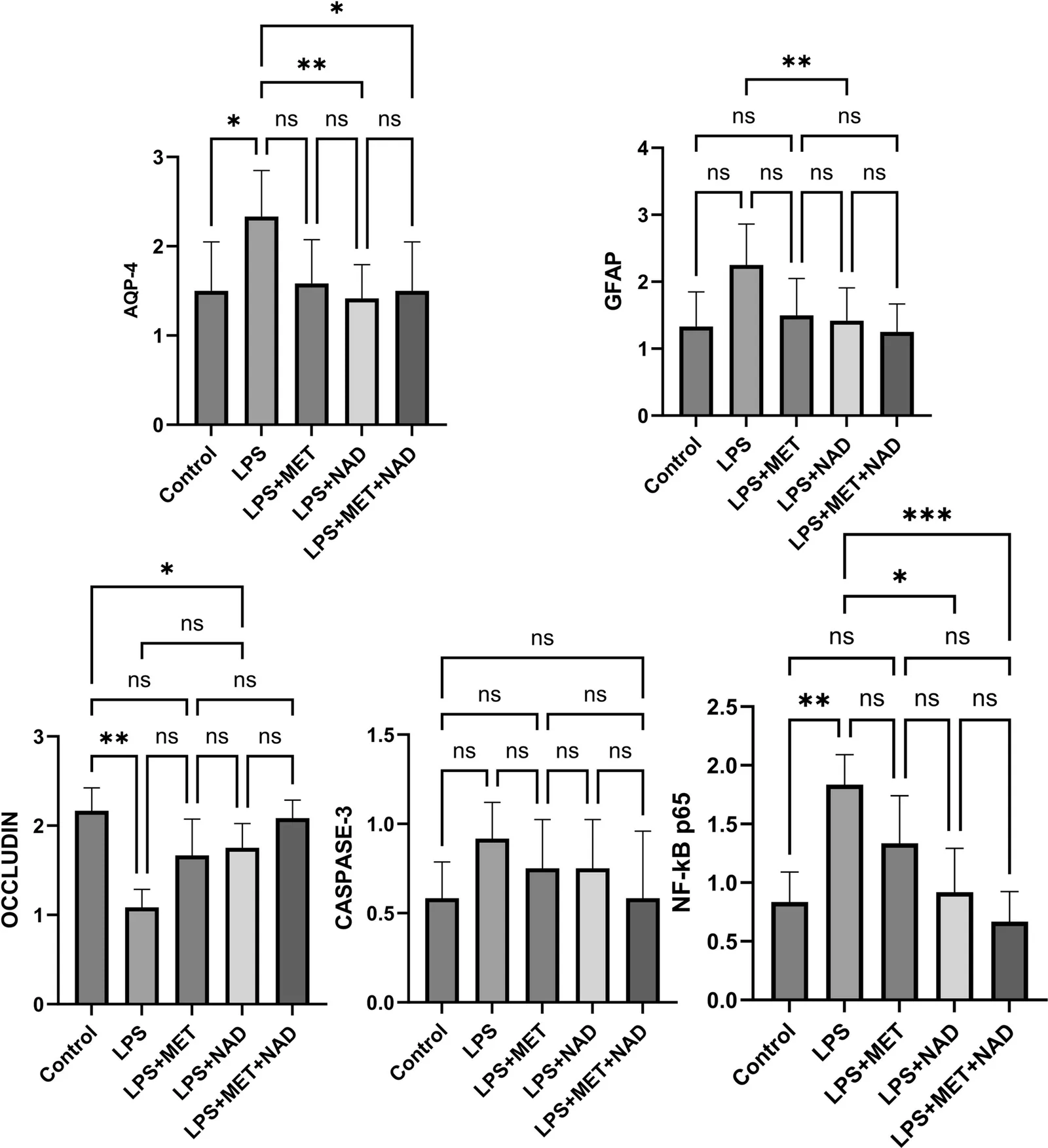
Quantitative analysis of immunohistochemical staining for all experimental groups. Bar graphs show semi-quantitative scoring of immunoreactivity for each marker. Values are presented as mean ± SD (*n* = 7) and analyzed using one-way ANOVA followed by Tukey’s test. Asterisks indicate statistically significant differences between groups (*p < 0.05, **p < 0.01, and ***p < 0.001)

The immunohistochemical evaluation of AQP-4, GFAP, Occludin, cleaved caspase-3, and NF-κB p65 expressions across all experimental groups is presented in Fig. 6, and their quantitative analyses are summarized in Fig. 7.

AQP-4 immunoreactivity was significantly elevated in the LPS group compared to the control group (*p* < 0.05). Treatment with methylene blue (LPS + MET), NAD⁺ (LPS + NAD), and the combination treatment (LPS + MET + NAD) led to a reduction in AQP-4 expression levels. Notably, LPS + NAD (*p* < 0.05) and LPS + MET + NAD (*p* < 0.01) significantly attenuated AQP-4 expression relative to LPS alone, indicating a potential additive effect (Fig. 6).

GFAP, a marker for astrocytic reactivity, demonstrated significantly increased immunoreactivity in the LPS group compared to controls (*p* < 0.01), suggestive of astrogliosis. MET and NAD⁺ treatments, individually or in combination, resulted in a decrease in GFAP-positive cells, although this reduction was only statistically significant in the LPS + NAD group (*p* < 0.01) compared to the LPS group (Fig. 6).

Occludin, a tight junction protein, was markedly reduced in the LPS group relative to control animals (*p* < 0.01), indicating disruption of the blood–brain barrier (BBB). While treatment groups (LPS + MET, LPS + NAD, and LPS + MET + NAD) exhibited a trend toward restored Occludin expression, only the LPS + NAD group showed a statistically significant improvement when compared to the LPS group (*p* < 0.05), suggesting a role for NAD in preserving BBB integrity (Fig. 6).

Cleaved caspase-3 expression, indicative of apoptosis, was slightly increased in the LPS group but did not differ significantly from the control group. Similarly, none of the treatment groups exhibited statistically significant changes in cleaved caspase-3 immunoreactivity compared to the LPS group, suggesting minimal apoptotic activity under the conditions tested (Fig. 6).

NF-κB p65, a key transcription factor involved in neuroinflammation, showed significantly increased nuclear immunoreactivity in the LPS group compared to controls (*p* < 0.01). Notably, methylene blue treatment (LPS + MET) led to a reduction in NF-κB p65 expression compared to the LPS group, while NAD⁺ alone (LPS + NAD) or in combination with methylene blue (LPS + MET + NAD) further attenuated this expression, with the combined treatment group showing a significant reduction (*p* < 0.001) (Fig. 6).

## Discussion

**Fig. 8:**
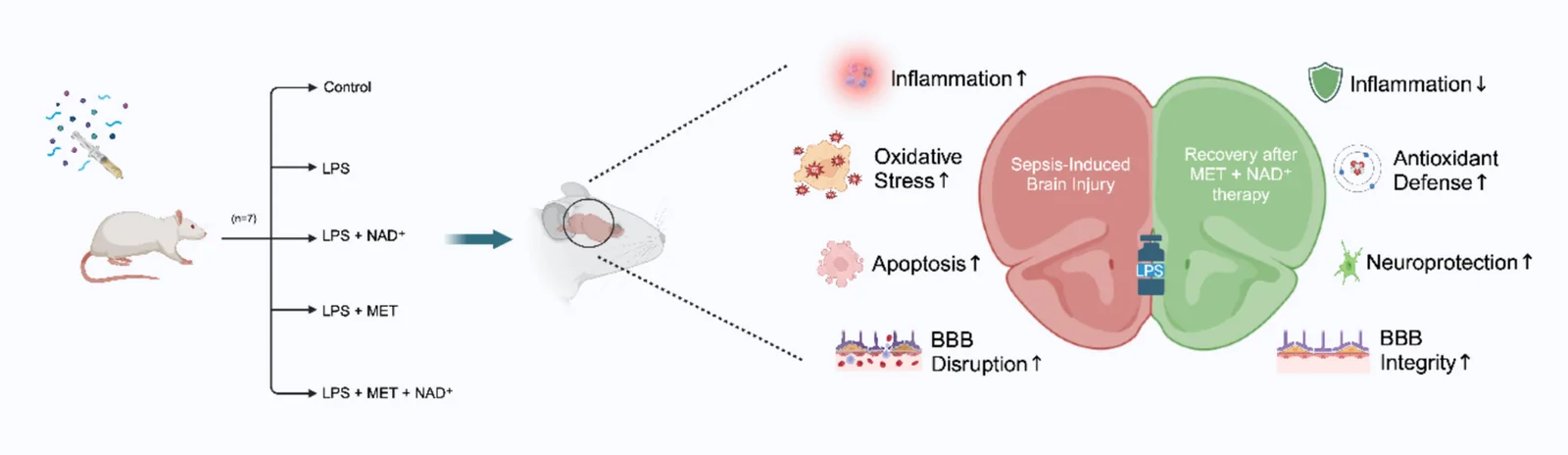
Mechanistic summary of MET and NAD⁺ treatment on LPS-induced brain injury

Sepsis-associated encephalopathy represents a severe complication affecting more than 50% of sepsis survivors, characterized by acute cognitive dysfunction and long-term neurological sequelae. The pathophysiology of SAE involves complex interactions between systemic inflammation, blood–brain barrier disruption, neuroinflammation, and oxidative stress (Sekino et al. 2022; Iwashyna et al. 2010; Liu et al. 2025, 2024). In the present study, we used an LPS-induced endotoxemia model to investigate acute neuroinflammatory responses. Although this model does not fully replicate clinical polymicrobial sepsis as it represents a single bolus intoxication rather than a true septic state with ongoing infection (Fink 2014; Lewis et al. 2016), it reliably reproduces key features of acute sepsis-associated brain injury, including elevated pro-inflammatory cytokines, microglial activation, and neuronal damage (Aydın and Bekmez 2023; Qin et al. 2023). Accordingly, our findings should be interpreted as reflecting acute inflammatory brain injury rather than the full clinical spectrum of SAE. As illustrated in the mechanistic summary (Fig.8), MET and NAD⁺ treatment counteracts LPS-induced brain injury through multiple complementary pathways, including suppression of neuroinflammation, enhancement of antioxidant defense, inhibition of apoptosis, and preservation of blood–brain barrier integrity.

Oxidative stress and inflammation are central to sepsis-induced brain injury, with myeloperoxidase (MPO) acting as both a marker and mediator (Xin et al. 2023; Pan et al. 2022). In sepsis, MPO promotes oxidant production and modulates inflammatory pathways, making it a potential therapeutic target (Pan et al. 2022; Chen et al. 2020). Our study showed that LPS significantly increased brain MPO activity, indicating enhanced neutrophil infiltration and oxidative stress, consistent with findings by Zarbato et al. who reported increased MPO activity in an experimental sepsis model (Zarbato et al. 2018). These findings are consistent with Wang et al., who reported increased MPO activity in a hindlimb ischemia–reperfusion model, and Demirbilek et al., who observed similar results in CLP-induced sepsis. MET, NAD, and especially their combination reduced MPO activity, with combination therapy normalizing levels. Our results also align with studies by Dinc et al. (Dinc et al. 2015)and Demirbilek et al., showing MET’s effectiveness in suppressing MPO activity in colitis and sepsis models (Demirbilek et al. 2006).

Our study demonstrated that combination therapy significantly increased SOD activity compared to single treatments, indicating an additive antioxidant effect. Wang et al. observed elevated SOD activity in lung tissue after MET treatment, and Demirbilek et al. reported increased SOD and catalase levels during sepsis (Demirbilek et al. 2006). The antioxidant role of NAD is supported by Hong et al., who showed that nicotinamide riboside elevated NAD levels and reduced oxidative stress in lung and heart tissues (Hong et al. 2018), and by Taheri et al., who found that nicotinamide mononucleotide upregulated glutathione peroxidase in cardiac tissue (Taheri et al. 2020). Consistent with our findings, Doganay et al. demonstrated that NAD⁺ administration attenuated acute hepatorenal oxidative injury and reduced pro-inflammatory cytokine levels in an experimental sepsis model, further supporting its protective role against sepsis-induced organ damage (Doganay 2022). The enhanced antioxidant capacity observed with the combination therapy may result from complementary mechanisms: MET reduces ROS via mitochondrial electron transport modulation (Tucker et al. 2018; Ballarin et al. 2024), while NAD supports redox balance through NAD-dependent enzymes like SIRT1. Hong et al. showed that SIRT1 inhibition negated NAD’s protective effects (Hong et al. 2018). Additionally, Umapati et al. demonstrated that β-NAD attenuated LPS-induced inflammation by reducing neutrophil infiltration and modulating cytokine expression (Umapathy et al. 2012).

Neuroinflammation plays a crucial role in sepsis-induced brain dysfunction (Pan et al. 2022). In our study, LPS level of LPS group increased IL-6, IL-8, TNF-α, and activated NF-κB in brain tissue. Both MET and NAD individually reduced these markers, supporting previous findings by Park et al. (Park et al. 2005), Fenn et al. (Fenn et al. 2015), and Li et al. (Li and Ying 2023)who reported anti-inflammatory effects of MET in septic shock, brain injury, and LPS-induced inflammation. Notably, the combination therapy showed a differential pattern across inflammatory markers: while NF-κB activation was reduced by all treatment groups, the combination therapy did not demonstrate superior cytokine suppression compared with individual treatments. In fact, MET and NAD monotherapies were more effective in reducing IL-6 levels than the combination, suggesting that co-administration may involve pharmacokinetic or mechanistic interactions that attenuate cytokine-specific responses. In a study, Memis et al. (Memis et al. 2002)reported a similar dissociation, where MET improved clinical parameters in sepsis without altering cytokine levels. Cross et al. (Cros et al. 2022)proposed that maintaining certain cytokines at moderate levels may enhance sepsis outcomes by shifting macrophages toward an anti-inflammatory phenotype, rather than blocking inflammation entirely. This idea is supported by Li et al. (Li et al. 2023) who found that NAD precursors reduced hippocampal inflammation and oxidative damage via the NAD/SIRT1 pathway.

One of the key findings of our study was the marked improvement in histopathological outcomes and BBB integrity with the combination therapy. LPS caused marked neuronal damage in the cortex, including necrosis, perineuronal vacuolization, vascular congestion, neuronophagy, and satellitosis. Both MET and NAD individually reduced these lesions, while their combination showed the greatest degree of histopathological improvement, indicating a complementary neuroprotective effect. This was supported by immunohistochemical data showing preserved tight-junction proteins and reduced neuroinflammation. Specifically, LPS increased AQP-4 and GFAP immunoreactivity while reducing occludin expression compared to controls, indicating BBB disruption and astrogliosis. Treatment with MET, NAD, or their combination reduced these pathological changes, with NAD and the combination therapy showing the most significant effects. Similar effects were reported by Ou et al. (Ou et al. 2023), who linked its anti-inflammatory effect to inhibition of the Siah2/Morg1/PHD3 pathway, and by Li et al. (Li et al. 2023) who reported suppression of STAT3 activation. Regarding BBB preservation, NAD treatment showed the most consistent effects, with statistically significant improvement in occludin expression (*p* < 0.05) and GFAP reduction (*p* < 0.01). The combination therapy further attenuated NF-κB p65 expression (*p*< 0.001), suggesting that each agent may contribute through partially overlapping mechanisms. Lomniczi et al. (Lomniczi et al. 2000)demonstrated that MET inhibits inducible nitric oxide synthase (iNOS) in the hypothalamus following LPS injection, preventing NO-mediated disruption of tight junctions. Concurrently, Alam et al. (Alam et al. 2019) found that nicotinamide treatment inhibited injury-induced activation of NF-κB and inflammatory mediators while preserving synaptic proteins in the brain.

The multifaceted neuroprotective effects of MET highlighted its action through multiple pathways across neurodegenerative disorders (Tucker et al. 2018). The normalization of occludin expression with combination therapy supports the role of BBB integrity in sepsis-associated encephalopathy. The enhanced efficacy of NAD in our study likely involves SIRT1 activation, as demonstrated by He et al. (He et al. 2024), who found that nicotinamide mononucleotide (NMN, an NAD precursor) ameliorated sepsis-induced acute lung injury through the SIRT1/NF-κB pathway, and by Su et al. (Su et al. 2024), who reported that NMN enhanced glutathione peroxidase 4 (GPX4)-mediated defense against LPS-induced ferroptosis in microglia, thereby inhibiting neuroinflammation.

The combination of MET and NAD is justified by their complementary mechanisms: MET has shown effectiveness in improving mitochondrial function and cognitive performance by functioning as an alternative mitochondrial electron carrier that bypasses sepsis-induced respiratory defects while also inhibiting nitric oxide synthase. NAD is crucial for cellular repair and energy production, and it has demonstrated promising effectiveness by activating sirtuin-dependent pathways that regulate oxidative stress and promote mitochondrial biogenesis (Quinlan et al. 2013; Verdin 2015). Both compounds possess established clinical safety profiles. MET demonstrates low toxicity at therapeutic doses with primary adverse effects being reversible blue discoloration and rare hemolytic anemia in G6PD-deficient patients (Smith 2025). NAD exhibits excellent safety with minimal gastrointestinal symptoms reported in clinical studies (Martens et al. 2018). Our combination therapy demonstrated superior antioxidant capacity and tissue protection compared to monotherapies, evidenced by enhanced SOD activity and reduced MPO levels. From a clinical perspective, both compounds are already approved for various indications (MET for methemoglobinemia and NAD precursors for neurological conditions), potentially facilitating translation (Yang et al. 2017; Johnson et al. 2018). Beyond sepsis, NAD precursors have shown consistent neuroprotective effects in other brain-injury models. Similarly, NAD administration after traumatic brain injury significantly reduced histological damage, brain edema, glial activation, and pro-inflammatory gene expression, largely via inhibition of the TLR2/4-NF-κB signaling pathway (She et al. 2025; Wei et al. 2017; Zhu et al. 2023). In addition, nicotinamide itself has been shown to exert protective effects against cerebral ischemia–reperfusion injury by reducing infarct size and preserving neurological function in a rat model (Arslan et al. 2024). These findings affirm that mitochondrial-targeting agents and NAD + precursors act through complementary pathways,including AMPK/PI3K-Akt, NF-κB/MAPK, and SIRT1/Nrf2 signaling across multiple types of brain injuries, further strengthening the rationale for combining methylene blue and NAD in sepsis-associated encephalopathy. The preserved blood–brain barrier integrity is particularly relevant, as BBB dysfunction correlates with poor neurological outcomes in sepsis patients. However, potential drug interactions warrant consideration, as both compounds could theoretically alter cytochrome P450-dependent pathways (Kox et al. 2000). The observation that combination therapy was less effective than individual treatments in reducing certain pro-inflammatory cytokines represents an important finding that should be considered when evaluating multi-target strategies. Possible explanations include pharmacokinetic interference between the two agents or opposing effects on specific signaling nodes, which warrant further mechanistic investigation. Given that 70% of sepsis survivors experience cognitive dysfunction (Iwashyna et al. 2010), our findings could inform adjunctive neuroprotective strategies to improve quality of life and reduce post-sepsis healthcare costs.

### Limitations

This study has several limitations that should be considered. First, the LPS-induced endotoxemia model represents a single bolus intoxication rather than a true septic state with ongoing infection, and therefore does not fully recapitulate the pathophysiology of clinical polymicrobial sepsis. Our findings should accordingly be interpreted as reflecting acute inflammatory brain injury rather than the full spectrum of SAE. Second, the 6-h observation period captures only the initial inflammatory response; later-stage events such as sustained microglial activation, progressive BBB disruption, and delayed neuronal degeneration could not be evaluated. In LPS models, inflammatory cytokines peak earlier and resolve faster than in polymicrobial models, where cytokine persistence better correlates with clinical disease severity. Third, while the combination therapy showed enhanced effects on oxidative stress markers and histopathological outcomes, individual treatments were more effective in reducing certain inflammatory cytokines, suggesting complementary rather than synergistic effects. Future studies using the CLP model with multiple time points and survival endpoints are warranted to better reflect the temporal progression and complexity of sepsis-associated brain injury.

## Conclusion

In conclusion, our study demonstrated that both MET and NAD provide neuroprotection against LPS-induced sepsis-related brain injury, with combination therapy yielding enhanced effects in several parameters. Treatment with these compounds significantly improved clinical manifestations, as assessed by Murine Sepsis Score, particularly with the combination therapy demonstrating the most pronounced reduction in overall disease severity. These treatments modulated oxidative stress (MPO, SOD), neuroinflammatory signaling (NF-κB, IL-6, IL-8, and TNF-α), and components of BBB (occludin, AQP-4) as well as glial activation (GFAP). While the combination therapy showed particular benefits in reducing MPO activity, enhancing SOD levels, and improving histopathological outcomes, individual treatments were more effective in reducing certain inflammatory cytokines. These findings suggest that targeting different pathophysiological mechanisms in sepsis-associated encephalopathy may offer therapeutic benefits, though optimal treatment strategies may depend on specific clinical contexts and therapeutic goals. Future investigations should explore prophylactic administration in high-risk surgical patients to prevent sepsis-associated encephalopathy development. Additionally, studies examining combination therapy effects on long-term neuroplasticity markers will determine whether enhanced mitochondrial function translates into sustained cognitive protection.

## Data Availability

The datasets used and/or analyzed during the current study are available from the corresponding author upon reasonable request.

## References

[CR1] Alam SI, Ur Rehman S, Ok Kim M (2019) Nicotinamide improves functional recovery via regulation of the RAGE/JNK/NF-κB signaling pathway after brain injury. J Clin Med. 10.3390/jcm8020271 30813383 10.3390/jcm8020271 PMC6406790

[CR2] Arslan R, Doganay S, Budak O, Bahtiyar N (2024) Investigation of preconditioning and the protective effects of nicotinamide against cerebral ischemia-reperfusion injury in rats. Neurosci Lett 840:137949 39181500 10.1016/j.neulet.2024.137949

[CR3] Aydın P, Bekmez H (2023) Experimental sepsis models: advantages and limitations. Eurasian J Med 55:S120 10.5152/eurasianjmed.2023.23364 PMC11075015 39109920

[CR4] Ballarin RS, Lazzarin T, Zornoff L, Azevedo PS, Pereira FWL, Tanni SE, Minicucci MF (2024) Methylene blue in sepsis and septic shock: a systematic review and meta-analysis. Front Med 11:1366062 10.3389/fmed.2024.1366062 PMC11063345 38698779

[CR5] Barichello T, Generoso JS, Collodel A, Petronilho F, Dal-Pizzol F (2021) The blood-brain barrier dysfunction in sepsis. Tissue Barriers 9:1840912 33319634 10.1080/21688370.2020.1840912 PMC7849782

[CR6] Borges A, Bento L (2024) Organ crosstalk and dysfunction in sepsis. Ann Intensive Care 14:147 39298039 10.1186/s13613-024-01377-0 PMC11413314

[CR7] Chen S, Chen H, Du Q, Shen J (2020) Targeting myeloperoxidase (MPO) mediated oxidative stress and inflammation for reducing brain ischemia injury: potential application of natural compounds. Front Physiol 11:433 32508671 10.3389/fphys.2020.00433 PMC7248223

[CR8] Collange O, Charles A-L, Bouitbir J, Chenard M-P, Zoll J, Diemunsch P, Thaveau F, Chakfé N, Piquard F, Geny B (2013) Methylene blue protects liver oxidative capacity after gut ischaemia–reperfusion in the rat. Eur J Vasc Endovasc Surg 45:168–175 23246335 10.1016/j.ejvs.2012.11.011

[CR9] Cros C, Margier M, Cannelle H, Charmetant J, Hulo N, Laganier L, Grozio A, Canault M (2022) Nicotinamide mononucleotide administration triggers macrophages reprogramming and alleviates inflammation during sepsis induced by experimental peritonitis. Front Mol Biosci 9:895028 35832733 10.3389/fmolb.2022.895028 PMC9271973

[CR10] Demirbilek S, Sizanli E, Karadag N, Karaman A, Bayraktar N, Turkmen E, Ersoy MO (2006) The effects of methylene blue on lung injury in septic rats. Eur Surg Res 38:35–41 16490992 10.1159/000091525

[CR11] Dinc S, Caydere M, Akgul G, Yenidogan E, Hucumenoglu S, Rajesh M (2015) Methylene blue inhibits the inflammatory process of the acetic acid-induced colitis in rat colonic mucosa. Int Surg 100:1364–1374 10.9738/INTSURG-D-15-00118.1 26062761

[CR12] Doganay S, Budak O, Sahin A, Bahtiyar N. (2022). Antioxidant and anti-inflammatory effects of nicotinamide adenine dinucleotide (NAD+) against acute hepatorenal oxidative injury in an experimental sepsis model. Kafkas Univ. Vet Fak Derg 28(1), 121–130.

[CR13] Fenn AM, Skendelas JP, Moussa DN, Muccigrosso MM, Popovich PG, Lifshitz J, Eiferman DS, Godbout JP (2015) Methylene blue attenuates traumatic brain injury-associated neuroinflammation and acute depressive-like behavior in mice. J Neurotrauma 32:127–138 25070744 10.1089/neu.2014.3514 PMC4291210

[CR14] Fink MP (2014) Animal models of sepsis. Virulence 5:143–153 24022070 10.4161/viru.26083 PMC3916368

[CR15] Guo J, Li B, Wang J, Guo R, Tian Y, Song S, Guo L (2021) Protective effect and mechanism of nicotinamide adenine dinucleotide against optic neuritis in mice with experimental autoimmune encephalomyelitis. Int Immunopharmacol 98:107846 34174704 10.1016/j.intimp.2021.107846

[CR16] Gureev AP, Sadovnikova IS, Popov VN (2022) Molecular mechanisms of the neuroprotective effect of methylene blue. Biochem Mosc 87:940–956 10.1134/S0006297922090073 36180986

[CR17] He S, Jiang X, Yang J, Wu Y, Shi J, Wu X, Du S, Zhang Y, Gong L, Dong S (2024) Nicotinamide mononucleotide alleviates endotoxin-induced acute lung injury by modulating macrophage polarization via the SIRT1/NF-κB pathway. Pharm Biol 62:22–32 38100537 10.1080/13880209.2023.2292256 PMC10732210

[CR18] Hong G, Zheng D, Zhang L, Ni R, Wang G, Fan G-C, Lu Z, Peng T (2018) Administration of nicotinamide riboside prevents oxidative stress and organ injury in sepsis. Free Radic Biol Med 123:125–137 29803807 10.1016/j.freeradbiomed.2018.05.073 PMC6236680

[CR19] Hotchkiss RS, Moldawer LL, Opal SM, Reinhart K, Turnbull IR, Vincent J-L (2016) Sepsis and septic shock. Nat Rev Dis Primers 2:1–21 10.1038/nrdp.2016.45 PMC5538252 28117397

[CR20] Iwashyna TJ, Ely EW, Smith DM, Langa KM (2010) Long-term cognitive impairment and functional disability among survivors of severe sepsis. JAMA 304:1787–1794 20978258 10.1001/jama.2010.1553 PMC3345288

[CR21] Ji M-h, Xia D-g, Zhu L-y, Zhu X, Zhou X-y, J-y X, Yang J-j (2018) Short-and long-term protective effects of melatonin in a mouse model of sepsis-associated encephalopathy. Inflammation 41:515–529 29198013 10.1007/s10753-017-0708-0

[CR22] Johnson S, Wozniak DF, Imai S (2018) CA1 Nampt knockdown recapitulates hippocampal cognitive phenotypes in old mice which nicotinamide mononucleotide improves. NPJ Aging Mech Dis 4:10 30416740 10.1038/s41514-018-0029-z PMC6224504

[CR23] Kara A, Ozkanlar S (2023) Blockade of P2X7 receptor‐mediated purinergic signaling with A438079 protects against LPS‐induced liver injury in rats. J Biochem Mol Toxicol 37:e23443 37365769 10.1002/jbt.23443

[CR24] Kox W, Volk T, Kox S, Volk H-D (2000) Immunomodulatory therapies in sepsis. Intensive Care Med 26:S124–S128 10786969 10.1007/s001340051129

[CR25] Kruger NJ. (2009). The bradford method for protein quantitation. In: Walker JM (ed) The protein protocols handbook. Springer Protocols Handbooks. Humana Press, Totowa, NJ. 10.1007/978-1-59745-198-7_4

[CR26] Kuperberg SJ, Wadgaonkar R (2017) Sepsis-associated encephalopathy: the blood–brain barrier and the sphingolipid rheostat. Front Immunol 8:597 28670310 10.3389/fimmu.2017.00597 PMC5472697

[CR27] Lewis AJ, Seymour CW, Rosengart MR (2016) Current murine models of sepsis. Surg Infect 17:385–393 10.1089/sur.2016.021 PMC4960474 27305321

[CR28] Li H-r, Liu Q, Zhu C-l, Sun X-y, Sun C-y, Yu C-m, Li P, X-m D, Wang J-f (2023) β-Nicotinamide mononucleotide activates NAD+/SIRT1 pathway and attenuates inflammatory and oxidative responses in the hippocampus regions of septic mice. Redox Biol 63:102745 37201414 10.1016/j.redox.2023.102745 PMC10206198

[CR29] Li Y, Ying W (2023) Methylene blue reduces the serum levels of interleukin-6 and inhibits STAT3 activation in the brain and the skin of lipopolysaccharide-administered mice. Front Immunol 14:1181932 37325623 10.3389/fimmu.2023.1181932 PMC10266349

[CR30] Liu H, Zhang T, Zhang L, Zhong Y (2025) Neuroinflammatory mechanisms of adult sepsis-associated encephalopathy: ımplications for blood–brain barrier disruption and oxidative stress. Diagnostics (Basel) 15:873 40218223 10.3390/diagnostics15070873 PMC11988331

[CR31] Liu Y, Hu S, Shi B, Yu B, Luo W, Peng S, Du X (2024) The role of iron metabolism in sepsis-associated encephalopathy: a potential target. Mol Neurobiol 61:4677–4690 38110647 10.1007/s12035-023-03870-2

[CR32] Lomniczi A, Cebral E, Canteros G, McCann SM, Rettori V (2000) Methylene blue inhibits the increase of inducible nitric oxide synthase activity induced by stress and lipopolysaccharide in the medial basal hypothalamus of rats. NeuroImmunoModulation 8:122–127 11124577 10.1159/000054271

[CR33] Mallah K, Couch C, Borucki DM, Toutonji A, Alshareef M, Tomlinson S (2020) Anti-inflammatory and neuroprotective agents in clinical trials for CNS disease and injury: where do we go from here? Front Immunol 11:2021 33013859 10.3389/fimmu.2020.02021 PMC7513624

[CR34] Martens CR, Denman BA, Mazzo MR, Armstrong ML, Reisdorph N, McQueen MB, Chonchol M, Seals DR (2018) Chronic nicotinamide riboside supplementation is well-tolerated and elevates NAD+ in healthy middle-aged and older adults. Nat Commun 9:1286 29599478 10.1038/s41467-018-03421-7 PMC5876407

[CR35] Mei B, Li J, Zuo Z (2021) Dexmedetomidine attenuates sepsis-associated inflammation and encephalopathy via central α2A adrenoceptor. Brain Behav Immun 91:296–314 33039659 10.1016/j.bbi.2020.10.008 PMC7749843

[CR36] Memis D, Karamanlioglu B, Yuksel M, Gemlik I, Pamukcu Z (2002) The influence of methylene blue infusion on cytokine levels during severe sepsis. Anaesth Intensive Care 30:755–762 12500513 10.1177/0310057X0203000606

[CR37] Moraes CA, Zaverucha-do-Valle C, Fleurance R, Sharshar T, Bozza FA, d’Avila JC (2021) Neuroinflammation in sepsis: molecular pathways of microglia activation. Pharmaceuticals (Basel) 14:416 34062710 10.3390/ph14050416 PMC8147235

[CR38] Ni J, Lin M, Jin Y, Li J, Guo Y, Zhou J, Hong G, Zhao G, Lu Z (2019) Gas6 attenuates sepsis-induced tight junction injury and vascular endothelial hyperpermeability via the Axl/NF-κB signaling pathway. Front Pharmacol 10:662 31263416 10.3389/fphar.2019.00662 PMC6585310

[CR39] Ou G, Che J, Dong J, Deng Y, Jiang X, Sun Y, He Z, Chen W, Zhang J (2023) Methylene blue targets PHD3 expression in murine microglia to mitigate lipopolysaccharide-induced neuroinflammation and neurocognitive impairments. Int Immunopharmacol 120:110349 37210913 10.1016/j.intimp.2023.110349

[CR40] Pan S, Lv Z, Wang R, Shu H, Yuan S, Yu Y, Shang Y (2022) Sepsis-induced brain dysfunction: pathogenesis, diagnosis, and treatment. Oxid Med Cell Longev 2022:1328729 36062193 10.1155/2022/1328729 PMC9433216

[CR41] Park B-K, Shim T-S, Lim C-M, Lee S-D, Kim W-S, Kim D-S, Kim W-D, Koh Y (2005) The effects of methylene blue on hemodynamic parameters and cytokine levels in refractory septic shock. Korean J Intern Med 20:123 16134766 10.3904/kjim.2005.20.2.123 PMC3891380

[CR42] Peng X, Luo Z, He S, Zhang L, Li Y (2021) Blood-brain barrier disruption by lipopolysaccharide and sepsis-associated encephalopathy. Front Cell Infect Microbiol 11:768108 34804998 10.3389/fcimb.2021.768108 PMC8599158

[CR43] Qin M, Gao Y, Guo S, Lu X, Zhao Q, Ge Z, Zhu H, Li Y (2023) Establishment and evaluation of animal models of sepsis-associated encephalopathy. World J Emerg Med 14:349 37908801 10.5847/wjem.j.1920-8642.2023.088 PMC10613796

[CR44] Quinlan CL, Perevoshchikova IV, Hey-Mogensen M, Orr AL, Brand MD (2013) Sites of reactive oxygen species generation by mitochondria oxidizing different substrates. Redox Biol 1:304–312 24024165 10.1016/j.redox.2013.04.005 PMC3757699

[CR45] Rajaee A, Barnett R, Cheadle WG (2018) Pathogen-and danger-associated molecular patterns and the cytokine response in sepsis. Surg Infect 19:107–116 10.1089/sur.2017.264 29364781

[CR46] Sekino N, Selim M, Shehadah A (2022) Sepsis-associated brain injury: underlying mechanisms and potential therapeutic strategies for acute and long-term cognitive impairments. J Neuroinflammation 19:101 35488237 10.1186/s12974-022-02464-4 PMC9051822

[CR47] She J, Zhang H, Xu H, Li Y-Y, Wu J-C, Han R, Lin F, Wang Y, Sheng R, Gu J-h (2025) Nicotinamide riboside restores nicotinamide adenine dinucleotide levels and alleviates brain injury by inhibiting oxidative stress and neuroinflammation in a mouse model of intracerebral hemorrhage. Mol Neurobiol 62:1321–1336 38981960 10.1007/s12035-024-04335-w PMC11772386

[CR48] Shrum B, Anantha RV, Xu SX, Donnelly M, Haeryfar SM, McCormick JK, Mele T (2014) A robust scoring system to evaluate sepsis severity in an animal model. BMC Res Notes 7:1–11 24725742 10.1186/1756-0500-7-233 PMC4022086

[CR49] Smith BA, Robinson R, Most AK (2025) From theory to therapy: methylene blue’s emerging role in the management of septic shock. J Pharm Pract 8971900251350554. 10.1177/08971900251350554 10.1177/08971900251350554 40492955

[CR50] Soliman H, Rawal B, Fulp J, Lee J-H, Lopez A, Bui MM, Khalil F, Antonia S, Yfantis HG, Lee DH (2013) Analysis of indoleamine 2–3 dioxygenase (IDO1) expression in breast cancer tissue by immunohistochemistry. Cancer Immunol Immunother 62:829–837 23344392 10.1007/s00262-013-1393-y PMC4501769

[CR51] Su R, Pan X, Chen Q, Wang J, Kong X, Li Y, Liu H, Hou X, Wang Y (2024) Nicotinamide mononucleotide mitigates neuroinflammation by enhancing GPX4-mediated ferroptosis defense in microglia. Brain Res 1845:149197 39216693 10.1016/j.brainres.2024.149197

[CR52] Sun Y, Koyama Y, Shimada S (2022) Inflammation from peripheral organs to the brain: how does systemic inflammation cause neuroinflammation? Front Aging Neurosci 14:903455 35783147 10.3389/fnagi.2022.903455 PMC9244793

[CR53] Taheri S, Arshadi S, Banaeifar A, Imanipour V (2020) The attenuating effect of high-ıntensity ınterval training and nicotinamide mononucleotide supplementation on aging-related oxidative stress in rat’s heart tissue. J Res Rehabil Sci 16:310–320

[CR54] Tucker D, Lu Y, Zhang Q (2018) From mitochondrial function to neuroprotection—an emerging role for methylene blue. Mol Neurobiol 55:5137–5153 28840449 10.1007/s12035-017-0712-2 PMC5826781

[CR55] Umapathy NS, Gonzales J, Fulzele S, Kim K-m, Lucas R, Verin AD (2012) β-Nicotinamide adenine dinucleotide attenuates lipopolysaccharide-induced inflammatory effects in a murine model of acute lung injury. Exp Lung Res 38:223–232 22563684 10.3109/01902148.2012.673049 PMC3678723

[CR56] Üstündağ H (2026) The P2X7 receptor/NLRP3 inflammasome signaling axis in sepsis: molecular mechanisms organ-specific pathophysiology and emerging therapeutic strategies. Purinergic Signal 22(2). 10.1007/s11302-026-10140-y 10.1007/s11302-026-10140-y PMC12972434 41803506

[CR57] Veldeman M, Coburn M, Rossaint R, Clusmann H, Nolte K, Kremer B, Höllig A (2017) Xenon reduces neuronal hippocampal damage and alters the pattern of microglial activation after experimental subarachnoid hemorrhage: a randomized controlled animal trial. Front Neurol 8:511 29021779 10.3389/fneur.2017.00511 PMC5623683

[CR58] Verdin E (2015) NAD+ in aging, metabolism, and neurodegeneration. Science 350:1208–1213 26785480 10.1126/science.aac4854

[CR59] Wei C-C, Kong Y-Y, Li G-Q, Guan Y-F, Wang P, Miao C-Y (2017) Nicotinamide mononucleotide attenuates brain injury after intracerebral hemorrhage by activating Nrf2/HO-1 signaling pathway. Sci Rep 7:717 28386082 10.1038/s41598-017-00851-z PMC5429727

[CR60] Xin Y, Tian M, Deng S, Li J, Yang M, Gao J, Pei X, Wang Y, Tan J, Zhao F (2023) The key drivers of brain injury by systemic inflammatory responses after sepsis: microglia and neuroinflammation. Mol Neurobiol 60:1369–1390 36445634 10.1007/s12035-022-03148-z PMC9899199

[CR61] Xie N, Zhang L, Gao W, Huang C, Huber PE, Zhou X, Li C, Shen G, Zou B (2020) NAD+ metabolism: pathophysiologic mechanisms and therapeutic potential. Signal Transduct Target Ther 5:227 33028824 10.1038/s41392-020-00311-7 PMC7539288

[CR62] Yakut S, Gelen V, Kara H, Özkanlar S, Yeşildağ A (2024) Silver nanoparticles loaded with oleuropein alleviates LPS-induced acute lung injury by modulating the TLR4/P2X7 receptor-mediated inflammation and apoptosis in rats. Environ Toxicol 39:4960–4973 38980228 10.1002/tox.24369

[CR63] Yang S-H, Li W, Sumien N, Forster M, Simpkins JW, Liu R (2017) Alternative mitochondrial electron transfer for the treatment of neurodegenerative diseases and cancers: Methylene blue connects the dots. Prog Neurobiol 157:273–291 26603930 10.1016/j.pneurobio.2015.10.005 PMC4871783

[CR64] Zarbato GF, de Souza Goldim MP, Giustina AD, Danielski LG, Mathias K, Florentino D, de Oliveira AN Junior, da Rosa N, Laurentino AO, Trombetta T (2018) Dimethyl fumarate limits neuroinflammation and oxidative stress and improves cognitive impairment after polymicrobial sepsis. Neurotox Res 34:418–430 29713994 10.1007/s12640-018-9900-8

[CR65] Zhu X, Cheng J, Yu J, Liu R, Ma H, Zhao Y (2023) Nicotinamide mononucleotides alleviated neurological impairment via anti-neuroinflammation in traumatic brain injury. Int J Med Sci 20:307 36860678 10.7150/ijms.80942 PMC9969499

